# A randomized trial of 13-Cis retinoic acid in children with advanced neuroblastoma after high-dose therapy

**DOI:** 10.1054/bjoc.2000.1425

**Published:** 2000-11

**Authors:** J A Kohler, J Imeson, C Ellershaw, S O Lie

**Affiliations:** Child Health, Southampton General Hospital, Southampton, SO16 6YD; United Kingdom Children’s Cancer Study Group, Department of Epidemiology and Public Health, University of Leicester, 22–28 Princess Road West, Leicester, LE1 6TP; Department of Pediatrics, The National Hospital, Pilestredet 32, Oslo, N-0027, Norway

**Keywords:** 13-Cis retinoic acid, neuroblastoma

## Abstract

One hundred and seventy-five children with Stage 3 or 4 neuroblastoma who had obtained a good response to conventional therapy were randomly allocated to 13-Cis retinoic acid at a dose of 0.75 mg/kg/day or placebo for up to 4 years. Toxicity was mild but no advantage in event-free survival was shown for the children receiving retinoic acid. © 2000 Cancer Research Campaign


*British Journal of Cancer* (2000) **83**(9), 1124–1127
© 2000 Cancer Research Campaign
doi: 10.1054/ bjoc.2000.1425, available online at http://www.idealibrary.com on IDEAL®

### A randomized trial of 13-Cis retinoic acid in children with advanced neuroblastoma after high-dose therapy

**JA Kohler[1], J Imeson[2], C Ellershaw[2] and SO Lie[3]***

[1]Child Health, Southampton General Hospital, Southampton, SO16 6YD; [2]United Kingdom Children's Cancer Study Group, Department of Epidemiology and Public Health, University of Leicester, 22–28 Princess Road West, Leicester, LE1 6TP; [3]Department of Pediatrics, The National Hospital, Pilestredet 32, N-0027, Oslo, Norway

**Summary** One hundred and seventy-five children with Stage 3 or 4 neuroblastoma who had obtained a good response to conventional therapy were randomly allocated to 13-Cis retinoic acid at a dose of 0.75 mg/kg/day or placebo for up to 4 years. Toxicity was mild but no advantage in event-free survival was shown for the children receiving retinoic acid. © 2000 Cancer Research Campaign

**Keywords:** 13-Cis retinoic acid; neuroblastoma

The prognosis for children with advanced stage neuroblastoma remains poor despite the increased intensity of treatment over the last 20 years. Many studies show that although 50–70% of patients have a very good response to chemotherapy, the majority subsequently relapse. This suggests the presence of minimal residual disease at cessation of therapy despite normal catecholamine secretion and no detectable signs of neuroblastoma by any method currently available. An alternative therapeutic approach is required to prevent these malignant cells from re-entering the growth cycle.

The in vitro differentiation of certain malignant cell lines including neuroblastoma can be induced by a variety of agents of which the retinoids are best studied (Thiele et al, 1985; Lippman et al, 1987). In vivo, retinoic acid has been shown to induce remission in promyelocytic leukaemias (Huang et al, 1988) and in xeroderma pigmentosum a 63% reduction in skin cancer was observed when patients received oral isotretinoin (Kraemer et al, 1988). Since 13-Cis retinoic acid has been used in children with xeroderma pigmentosum for 2 years with acceptable side-effects this retinoid at a similar low dose was chosen for this study. The aim of the study was to establish whether 13-Cis retinoic acid, used as continuation therapy after obtaining a good response to conventional chemotherapy, could prolong disease free survival in children with advanced neuroblastoma.

### PATIENTS

175 patients (Table 1) from 10 countries (England, Scotland, Wales, Belgium, Spain, South Africa, Norway, Sweden, Denmark, Netherlands) were recruited into the study from 1989 to 1997. All were in complete remission or very good partial remission following chemotherapy and were initially Stage 3 or Stage 4 neuroblastoma. Patients from the UK, Belgium and Scandinavian countries (143 of the 175 entered) followed ENSG protocols with chemotherapy comprising Vincristine, Carboplatin/Cisplatin, Etoposide and Cyclophosphamide. The protocols did not include radiotherapy, and high dose Melphalan was recommended for children with Stage 4 disease over the age of one year, and stage 3 disease with MYCN amplification (where available). Spanish patients received the same drugs during induction therapy, but high dose therapy comprised BCNU and VM26 in addition to melphalan. $I^{131}$ mIBG therapy preceded similar chemotherapy for patients from the Netherlands, and none of the three South African patients received high dose chemotherapy. Only patients in first remission were eligible for this study. In all, 126 patients received high dose melphalan and a transplant and the median time from diagnosis to the start of 13-Cis retinoic acid therapy was 341 days (lower quartile 289 days, upper quartile 406 days).

The trial was a double-blind randomized study and randomization was stratified for age (less than or greater than 1 year), treatment centre and complete response (CR) versus very good partial response (VGPR). INSS staging and response criteria were used (Brodeur et al, 1988). 13-Cis retinoic acid or identical placebo capsules were given once daily at a dose of 0.75 mg/kg/day with a milky drink or fat-containing meal. The patients were reviewed, initially monthly, to check for disease progression or signs of retinoic acid toxicity. The medication was prescribed for 4 years or until relapse.

Participating centres obtained protocol approval from the relevant research ethics committees and the randomization procedure was carried out by the UKCCSG data centre. Twice yearly progress reports on all patients were requested.

### STATISTICAL METHODS

The primary analysis evaluated treatment efficacy by comparison of all patients in the groups to which they were randomly allocated – an intention to treat analysis (Peto et al, 1976). The main endpoint used to evaluate the treatment efficacy was probability of event-free survival (EFS). This time was defined as the length of

*Accepted 12 October 1999*
*Revised 12 May 2000*
*Accepted 8 July 2000*

*Correspondence to:* JA Kohler

*On behalf of the European Neuroblastoma Study Group

**Table 1** Patient characteristics

| | RA | Placebo |
|---|---|---|
| Total no | 88 | 87 |
| Under 1 year | 5 | 8 |
| Over 1 year | 83 | 79 |
| CR | 59 | 60 |
| VGPR | 29 | 27 |
| UK centres | 60 | 60 |
| Non-UK centres | 28 | 27 |
| Stage 3 | 11 | 17 |
| Stage 4 | 77 | 70 |

time from randomization to earliest detection of relapse or progression, or death from any cause. Surviving relapse-free patients were censored at the date of the last clinical follow-up. The survival curves were calculated by standard methods (Parmar and Machin, 1995) and confidence intervals calculated. Univariate analyses were carried out using the log-rank test to evaluate treatment efficacy.

Multivariate analyses were carried out using a proportional hazards model to estimate the relative benefit of treatment after adjustment for stage, age greater or less than one year, and abdominal primary tumour or not in a forward stepwise regression.

### RESULTS

At the time of analysis in April 1999 median follow-up was 59 months (range 13 to 109 months). 102 patients had died of whom one died of a second malignancy and one of cerebral haemorrhage when thrombocytopenic following autografting. All other deaths were due to relapsed or progressive neuroblastoma. 5 children were still alive following relapse (i.e. 107 events). 16 patients (9 on placebo, 7 on RA) had completed 4 years treatment.

EFS of all patients is 39.6% at 3 years from randomization (95% C.I., 32.6% to 47.1%) and 37.3% at 5 years. Survival (S) for all patients is 42.8% at 3 years from randomization (95% C.I., 35.5% to 50.4%) and 39.7% at 5 years.

When treatment groups were compared no clear difference in EFS emerged, 3 year EFS for patients receiving RA 37% vs. 42% for patients receiving placebo, log-rank test *P* value 0.62 (Figure 1). After adjustment for prognostic factors: age under 1, abdominal primary and bone marrow metastases, the estimated relative treatment benefit remained similar (Hazard ratio 0.95, *P* value 0.8). No apparent advantage emerged in high risk patients: for Stage 4 over the age of 1 year overall 3 year EFS was 32.2%. For patients receiving RA it was 29% vs. 36% for patients receiving placebo, log-rank test *P* value 0.35 (Figure 2).

Compliance with treatment, assessed by parental reporting, was a problem since the capsules were large and median age at randomization was 3.5 years (range 0.4 to 21.2 years). The median time from high dose therapy to randomization was 67 days (lower quartile 42.5 days, upper quartile 99 days). There was then a delay of median 1 month before treatment could commence, of similar length for both treatment arms. The median time from diagnosis to randomization was 307 days (lower quartile 255 days, upper quartile 382 days). Six patients relapsed before treatment started, within 2 months of randomization, casting doubt on remission status at randomization. Four further patients relapsed within 2 months of randomization but had started treatment. 20 patients

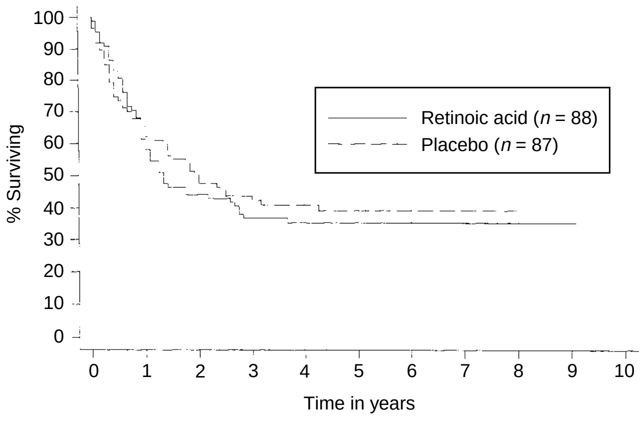

**Figure 1** EFS by treatment for all patients

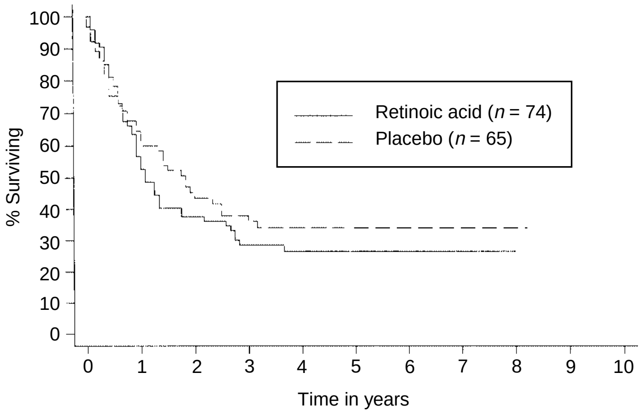

**Figure 2** EFS by treatment for stage 4 over the age of 1 year patients

**Table 2** Reported toxicity

| | RA | Placebo |
|---|---|---|
| Dry skin | 47 | 29 |
| Cheilitis | 24 | 9 |
| Bone pain | 16 | 16 |
| Other | 13 | 6 |

took treatment for less than 2 months from starting the first course, 5 because of early relapse and 15 were unable to take capsules for a variety of reasons. Omitting all 30 patients (of whom 15 were taking RA and 15 placebo) in a compliance analysis again showed no benefit in terms of 3 year EFS, for patients receiving RA it was 46% vs. 43% patients receiving placebo, log-rank test *P* value 0.91 (Figure 3). Re-defining non-compliance as failure to take capsules for 2 months unless relapsing did not materially change the result.

Toxicity, assessed clinically, was mild and comprised dry skin, cheilitis or bone pain, reported in both arms of the trial (Table 2). Treatment was discontinued because of presumed toxicity in only 5 cases. One child had recurrent skin problems and one bone pain, both were subsequently found to be on retinoic acid. Two children, both subsequently found to be on placebo, had eye symptoms and a further child on placebo stopped medication because of slow blood count recovery following high dose chemotherapy. Since most children had their central venous lines removed after

© 2000 Cancer Research Campaign

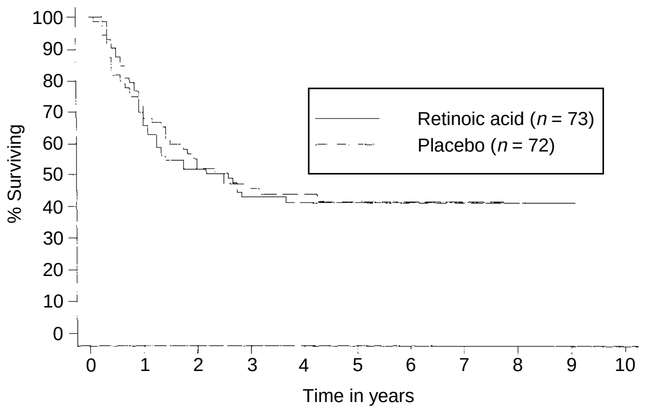

**Figure 3** EFS by treatment for compliant patients completion of standard chemotherapy, blood tests to check fasting triglyceride levels and liver enzymes were not compulsory. However, these were requested if blood was being taken for other reasons, or if the child had clinical signs of toxicity. They were normal in 35 and abnormal in 9 children tested.

### DISCUSSION

In the mid-1980s the only retinoid available for clinical use was 13-Cis retinoic acid. Based on experience with this agent given in other diseases, such as Xeroderma pigmentosum, the ENSG chose a dose designed to cause minimal side-effects since most of these patients had been through many months of extremely intensive treatment. They were unlikely to tolerate further discomfort for an experimental medication when the long-term prognosis was poor.

Whilst this trial has been in progress, further studies have been published showing 13-Cis retinoic acid to be a potent inducer of differentiation of neuroblastoma in vivo (Reynolds et al, 1991) and to induce clinical responses in some patients with assessable disease (Finklestein et al, 1992). A phase 1 trial was carried out by the Children's Cancer Group (Villablanca et al, 1995) in patients who had recently completely myeloablative therapy supported by bone marrow transplantation. Using a pulsed high dose schedule, 13-Cis retinoic acid was dose-escalated to a maximally tolerated dose of 160 mg/m$^2$/day with peak drugs levels of 7 μM.

This led to a phase III trial in which 13-Cis retinoic acid was given to similar children at 160 mg/m$^2$/day for two weeks per month for 6 months (Matthay et al, 1999). This was approximately 8 times the daily dose used in the ENSG study, although in the latter the medication was used continuously over a 4-year period. High risk patients were more precisely defined in the CCG study in terms of biology and histology. The results showed a 3-year EFS rate (from randomization after myeloablative therapy) of 46% for patients assigned to receive 13-Cis retinoic acid which was significantly better than the rate of 29% for those assigned to no further therapy ($P$ = 0.027). Our 3 year EFS rate for all patients of 39.6% (37% RA vs 42% placebo) may reflect not only sub-optimal 13-Cis retinoic acid levels, but the inclusion of some 'better risk' patients.

If only Stage 4 patients over the age of one year are considered, however, the CCG found a 3 year EFS of 40% for those assigned to 13-Cis retinoic acid compared with 25% for those assigned to no further therapy which was not a significant difference ($P$ =0.09). Our ENSG figures are 29% and 36% respectively ($P$ =0.35). If only the sub-group of Stage 4 patients who were in complete remission at the end of initial therapy were included in the CCG analysis, the improved outcome for those receiving retinoic acid became significant ($P$ = 0.03). In our ENSG study, the numbers become too small if those in compete remission are sub-divided from those in very good partial remission. It may also be that analysing the 3-year event-free survival is premature, and we must revisit these data in the future.

There are theoretical reasons for prescribing other synthetic retinoids. 9 Cis-retinoic acid has been shown to be a more effective differentiating agent in vitro (Lovat et al, 1997), but small clinical trials in adult patients suggest unacceptable toxicity, particularly headaches (Miller et al, 1995). Another derivative, fenretinide, is currently undergoing phase I studies in Italy and the United States.

This study commenced at a time when important prognostic factors such as MYCN gene amplification and chromosome 1p deletion were not widely available in ENSG countries. Definition of high risk patients was therefore sub-optimal and stratification for these factors not possible. Nevertheless these factors should be in approximate balance by virtue of randomization, and a separate analysis of accepted high risk patients, those over the age of one year with Stage 4 disease, was carried out.

This study has shown that 13-Cis retinoic acid can be given continuously at low dosage for a prolonged period of time to children with neuroblastoma, but that it does not affect the event-free survival. The higher dose intermittent schedule used by the CCG is currently recommended for children in complete response after initial therapy.

### ACKNOWLEDGEMENTS

The capsules were supplied by Roche International and distributed by the Norwegian Medicinal Depot.

### Participating centres

Royal Aberdeen Children's Hospital
St Bartholomew's Hospital, London
Birmingham Children's Hospital
Bristol Royal Hospital for Sick Children
Llandough Hospital, Cardiff
Royal Hospital for Sick Children, Glasgow
Great Ormond Street Hospital, London
St James' University Hospital, Leeds
Royal Manchester Children's Hospital
Royal Victoria Infirmary, Newcastle
Royal Marsden NHS Trust, London
Sheffield Children's Hospital
Southampton General Hospital
Emma Kinderziekenhuis, Amsterdam
A.Z. Kinderen VUB, Brussels
University Hospital, Leuven
National University Hospital, Copenhagen
University of Pretoria, South Africa
Hospital Infantil La Fe, Valenica
Hospital La Paz, Madrid
University Children's Hospital, Lund
East Hospital, Gothenburg
Rikshospitalet, Oslo
Haukeland Hospital, Bergen

*© 2000 Cancer Research Campaign*

### REFERENCES

Brodeur GM, Seeger RC, Barrett A, Berthold F, Castleberry RF, D'Angio G, De Bernardi B, Evans AE, Favrot M and Freeman AI (1988) International criteria for diagnosis, staging and response to treatment in patients with neuroblastoma. *J Clin Oncol* **12**: 1874–1881

Finklestein JZ, Krailo MD, Lenarsky C, Ladison S, Blair G, Reynolds CP, Sitarz AL and Hammond GD (1992) 13-*Cis*-retinoic acid (NSC 122758) in the treatment of children with metastatic neuroblastoma unresponsive to conventional chemotherapy: Report from the Children's Cancer Study Group. *Med Pediatr Oncol* **20**: 307–311

Huang ME, Ye YC, Chen SR, Chai JR, Lu JX, Zhon L, Gu LJ and Wang ZY (1988) Use of all-trans retinoic acid in the treatment of acute promyelocytic leukaemia. *Blood* **72**: 567–572

Kraemer KH, DiGiovanna JJ, Moshell AN, Tarone RE and Peck GL (1988) Prevention of skin cancer in xeroderma pigmentosum with the use of oral Isotretinoin. *N Engl J Med* **318**: 1633–1637

Lippmann SM, Kessler JF and Meyskens FL Jr (1987) Retinoids as preventative and therapeutic anti-cancer agents. *Cancer Treat Rep* **71**: 391–405

Lovat PE, Irving H, Malcolm AJ, Pearson AD and Redfern CP (1997) 9 Cis-retinoic acid – a better retinoid for the modulation of differentiation, proliferation and gene expression in human neuroblastoma. *J Neurooncol* **31**: 85–91

Matthay KK, Villablanca JG, Seeger RC, Stram DO, Harris RE, Ramsay NK, Swift P, Shimada H, Black CT, Garrett M, Brodeur MD, Gerbing RB and Reynolds CP (1999) Treatment of high risk neuroblastoma with intensive chemotherapy, radiotherapy, autologous bone marrow transplantation, and 13-Cis-retinoic acid. *N Eng J Med* **341**: 1165–1173

Miller WH Jr, Jakubowski A, Tong WP, Miller VA, Rigas JR, Benedetti F, Gill GM, Truglia JA, Ulm E, Shirley M and Warren RP Jr. (1995) 9-Cis retinoic acid induces complete remission but does not reverse clinically acquired retinoid resistance in acute promyelocytic leukaemia. *Blood* **85**: 3021–3027

Parmar MKB and Machin D (1995) *Survival Analysis: A Practical Approach*. John Wiley & Sons; Chichester

Peto R, Pike MC, Armitage P, Breslow NE, Cox DR, Howard SV, Mantel N, McPherson K, Peto J and Smith PG (1976) Design and analysis of randomised clinical trials requiring prolonged observation of each patient. 1. Introduction and Design. *Br J Cancer* **34**: 585–612

Reynolds CP, Kane DJ, Einhorn PA, Matthay KK, Crouse VL, Wilbur JR, Shurin SB and Seeger RC (1991) Response of neuroblastoma to retinoic acid in vitro and in vivo. *Prog Clin Biol Res* **366**: 203–211

Thiele CJ, Reynolds CP and Israel MA (1985) Decreased expression of N-myc preceded retinoic acid – induced morphological differentiation of human neuroblastoma. *Nature* **313**: 404–406

Villablanca JG, Khan AA, Avramis VI, Seeger RC, Matthay KK Ramsay NK and Reynolds CP (1995) Phase 1 trial of 13-*Cis*-retinoic acid in children with neuroblastoma following bone marrow transplantation. *J Clin Oncol* **13**: 894–901

© 2000 Cancer Research Campaign
